# Immunohistochemistry and oxygen saturation endoscopic imaging reveal hypoxia in submucosal invasive esophageal squamous cell carcinoma

**DOI:** 10.1002/cam4.6217

**Published:** 2023-06-17

**Authors:** Nobuhisa Minakata, Shingo Sakashita, Masashi Wakabayashi, Yuka Nakamura, Hironori Sunakawa, Yusuke Yoda, Genichiro Ishii, Tomonori Yano

**Affiliations:** ^1^ Department of Gastroenterology and Endoscopy National Cancer Center Hospital East Kashiwa Japan; ^2^ Course of Advanced Clinical Research of Cancer Juntendo University Graduate School of Medicine Bunkyo‐ku Japan; ^3^ Division of Pathology Exploratory Oncology Research and Clinical Trial Center, National Cancer Center Kashiwa Japan; ^4^ Biostatistics Division, Center for Research Administration and Support National Cancer Center Kashiwa Japan; ^5^ Department of Strategic Programs Exploratory Oncology Research and Clinical Trial Center, National Cancer Center Kashiwa Japan; ^6^ NEXT Medical Device Innovation Center National Cancer Center Hospital East Kashiwa Japan; ^7^ Department of Gastroenterology and Endoscopy Saitama Cancer Center Saitama Japan; ^8^ Department of Pathology and Clinical Laboratories National Cancer Center Hospital East Kashiwa Japan

**Keywords:** blood vessel, endoscopy, esophageal squamous cell carcinoma, hypoxia, neoplasm invasion

## Abstract

**Background:**

Hypoxic microenvironment is prominent in advanced esophageal squamous cell carcinoma (ESCC). However, it is unclear whether ESCC becomes hypoxic when it remains in the mucosal layer or as it invades the submucosal layer. We aimed to investigate whether intramucosal (Tis‐T1a) or submucosal invasive (T1b) ESCC becomes hypoxic using endoscopic submucosal dissection samples.

**Methods:**

We evaluated the expression of hypoxia markers including hypoxia inducible factor 1α (HIF‐1α), carbonic anhydrase IX (CAIX), and glucose transporter 1 (GLUT1) by H‐score and vessel density by microvessel count (MVC) and microvessel density (MVD) for CD31 and α‐smooth muscle actin (α‐SMA) with immunohistochemical staining (*n* = 109). Further, we quantified oxygen saturation (StO_2_) with oxygen saturation endoscopic imaging (OXEI) (*n* = 16) and compared them to non‐neoplasia controls, Tis‐T1a, and T1b.

**Results:**

In Tis‐T1a, cccIX (13.0 vs. 0.290, *p* < 0.001) and GLUT1 (199 vs. 37.6, *p* < 0.001) were significantly increased. Similarly, median MVC (22.7/mm^2^ vs. 14.2/mm^2^, *p* < 0.001) and MVD (0.991% vs. 0.478%, *p* < 0.001) were markedly augmented. Additionally, in T1b, the mean expression of HIF‐1α (16.0 vs. 4.95, *p* < 0.001), CAIX (15.7 vs. 0.290, *p* < 0.001), and GLUT1 (177 vs. 37.6, *p* < 0.001) were significantly heightened, and median MVC (24.8/mm^2^ vs. 14.2/mm^2^, *p* < 0.001) and MVD (1.51% vs. 0.478%, *p* < 0.001) were markedly higher. Furthermore, OXEI revealed that median StO_2_ was significantly lower in T1b than in non‐neoplasia (54% vs. 61.5%, *p* = 0.00131) and tended to be lower in T1b than in Tis‐T1a (54% vs. 62%, *p* = 0.0606).

**Conclusion:**

These results suggest that ESCC becomes hypoxic even at an early stage, and is especially prominent in T1b.

## Graphical abstract

Immunohistochemistry and oxygen saturation endoscopic imaging reveal that it is markedly hypoxic in submucosal invasive esophageal squamous cell carcinoma (ESCC) and close to normoxia in intramucosal ESCC. This is significant because it clarifies the biological characteristics of the oxygen environment in ESCC. In addition, oxygen saturation may be more useful in the prediction of the depth of invasion in superficial ESCC.

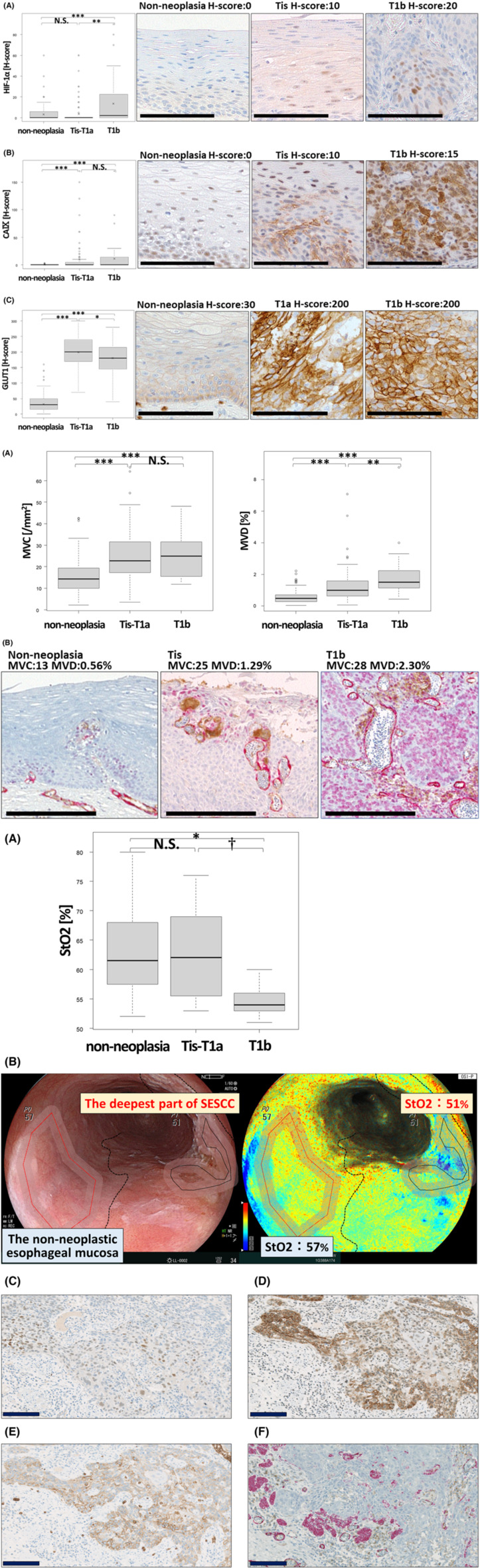

## 1 INTRODUCTION

In advanced solid cancers, it is known that the abnormal growth of cancer cells and incomplete angiogenesis can lead to tumor hypoxia.^1^, ^2^, ^3^ In the absence of oxygen, hypoxia inducible factor 1α (HIF‐1α), which is the hypoxic response factor, remains stable, activating the expression of hypoxia response genes, such as carbonic anhydrase IX (*CAIX*),^4^ glucose transporter 1 (*GLUT1*),^5^ vascular endothelial growth factor (*VEGF*),^3^, ^6^, ^7^ erythropoietin (*EPO*),^3^ transforming growth factor beta 3 (*TGF‐β3*),^3^ and so on. This leads to further angiogenesis, tumor invasion, and metastasis, resulting in a poor prognosis.^2^, ^3^, ^4^, ^5^, ^8^ As a result of angiogenesis, the density and number of blood vessels increase in the tumor.^3^, ^9^, ^10^

Esophageal squamous cell carcinoma (ESCC) is one of the tumors that becomes hypoxic as it progresses, which leads to angiogenesis. It has a poor prognosis and high mortality rate.^9^, ^10^, ^11^, ^12^, ^13^, ^14^, ^15^, ^16^, ^17^, ^18^, ^19^ In non‐neoplastic esophageal tissues, the expression of hypoxia markers such as HIF‐1α, CA9, and GLUT1 is rare,^20^, ^21^, ^22^ but in ESCC, this expression increases as it invades deeper, and the high expression of hypoxia markers is associated with a poor prognosis.^10^, ^12^, ^13^, ^14^, ^15^, ^16^, ^17^

In superficial esophageal squamous cell carcinoma (SESCC), it has been reported that the expression of hypoxia markers is higher than that in non‐neoplasia^20^, ^21^, ^22^ and lower than that in advanced ESCC.^10^, ^12^, ^13^, ^14^, ^15^, ^16^, ^17^ Furthermore, oxygen saturation endoscopic imaging (OXEI, FUJIFILM, Tokyo) collaboratively developed by our group, for visualizing the oxygen status of digestive tract lesions as a color map in real time^23^, ^24^, ^25^ has previously revealed that the oxygen saturation (StO_2_) in esophageal neoplasia was significantly lower than that in non‐neoplasia.^23^ This finding is consistent with previous reports relating to the expression of hypoxia markers.^10^, ^12^, ^13^, ^14^, ^15^, ^16^, ^17^, ^20^, ^21^, ^22^

Recent literature suggests that ESCC is hypoxic at an early stage. However, there have been no reports comparing the oxygen status of non‐neoplasia, high‐grade dysplasia (pTis), intramucosal carcinoma (pT1a), and submucosal carcinoma (pT1b). Further, it is also unknown whether ESCC is hypoxic when it remains in the mucosal layer or whether it becomes hypoxic as it invades the submucosal layer.

The aim of this study was therefore to investigate whether ESCC becomes hypoxic in pTis‐T1a or pT1b by comparing the expression of hypoxia markers, vessel density, and the StO_2_ with OXEI in non‐neoplasia, pTis‐T1a, and pT1b, respectively.

## 2 MATERIALS AND METHODS

### 2.1 Patients

Patients with SESCC who underwent endoscopic submucosal dissection (ESD) at the National Cancer Center Hospital East, with histologically diagnosed negative horizontal and vertical margins, and with the pathological staging of pTis or pT1a between March 2019 and October 2019 and with the pathological staging of pT1b between September 2015 and October 2019, met the inclusion criteria of this study. Patients who had a history of chemotherapy and/or radiotherapy for esophageal and/or head and neck cancers were excluded from the study.

### 2.2 Histopathologic examinations

Routine pathologic diagnoses of ESD specimens were performed as follows: Each ESD specimen was fixed in 10% neutral buffered formalin and cut into 2 mm slices, vertical to the long axis, and then processed for paraffin embedding and sectioning. Hematoxylin and eosin staining (H&E) was performed, and then the specimens were examined using light microscopy by two pathologists. The depth of invasion, lateral and deep margins, degree of differentiation, and lymphatic and vascular invasion were reported based on the Japanese Classification of Esophageal Cancer, and the T classification was categorized according to the guidelines of the 8th edition of the TNM classification.^26^, ^27^

### 2.3 Immunohistochemical staining

Immunohistochemical staining was performed on the VENTANA BenchMark ULTRA automated slide stainer (VENTANA, Roche). The primary antibodies used in this study are listed in Table S1.

### 2.4 Evaluation of the expression of HIF‐1α, CAIX, and GLUT1

**FIGURE 1:**
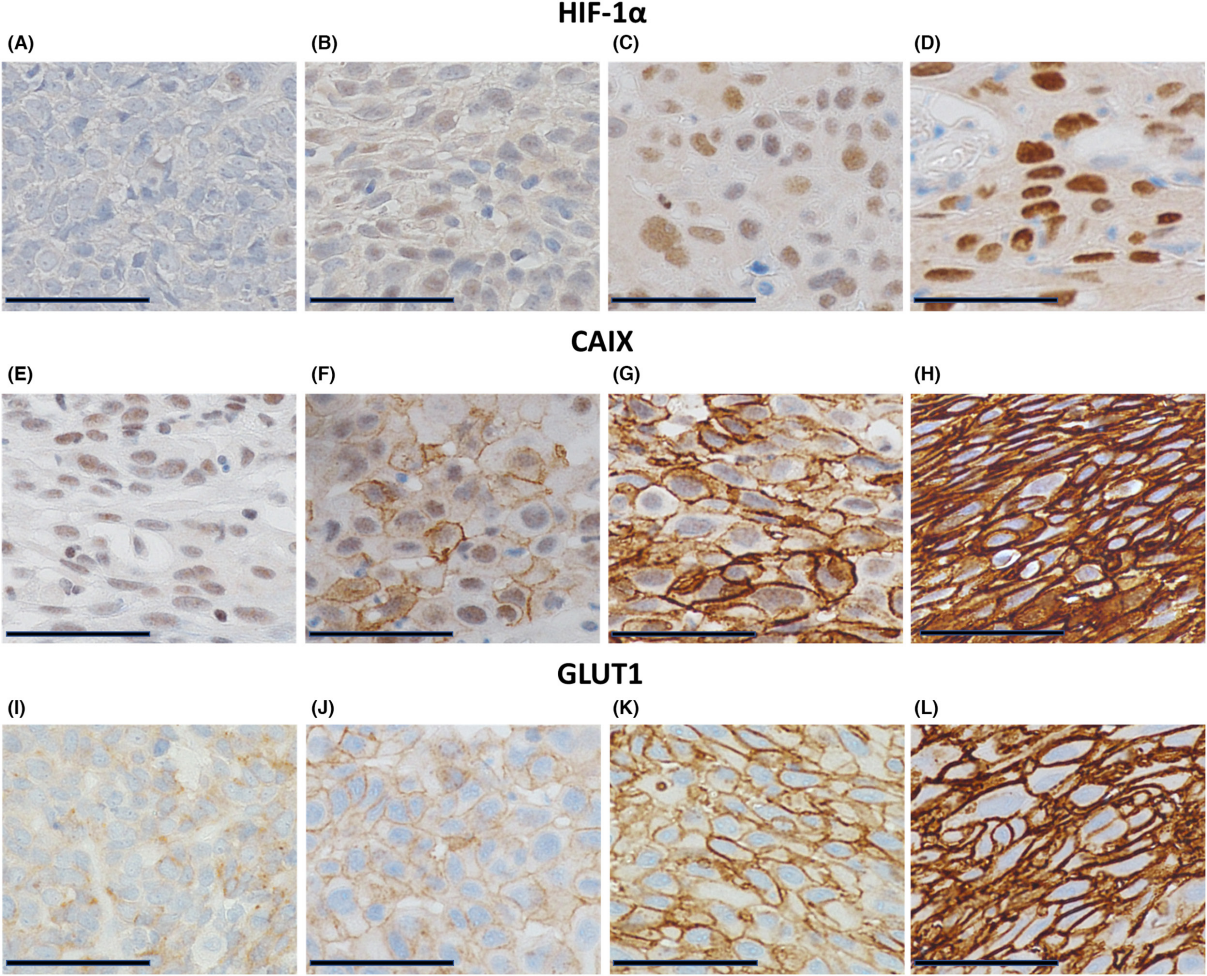
(A–L) Representative photomicrographs of immunohistochemical staining of HIF‐1α (A–D), CAIX (E–H), and GLUT1 (I–L). The intensity of staining was evaluated in the nuclei for HIF‐1α and in the membrane for CAIX and GLUT1. Scale Bar: 50 μm. (A, E, I) 0, negative staining. (B, F, J) 1+, weak staining. (C, G, K) 2+, moderate staining. (D, H, L) 3+, strong staining.

The SESCC and the adjacent non‐neoplastic esophageal epithelium, in one target section with the most widespread cancer cells in pTis‐T1a or with the deepest depth of invasion by cancer cells in pT1b, together with the expression of HIF‐1α, CAIX, and GLUT1 were evaluated with immunohistochemical staining, and H‐score assignment was performed by two pathologists.^28^, ^29^, ^30^ The staining intensity of HIF‐1α based on nuclear staining and that of CAIX and GLUT1 based on membrane staining was scored using the following classification system: [0], negative staining; [1+], weak staining; [2+], moderate staining; [3+], strong staining (Figure 1). Thus, the H‐score was calculated as follows: 1 × (the percentage of cancer cells staining [1+]) + 2 × (the percentage of cancer cells staining [2+]) + 3 × (the percentage of cancer cells staining [3+]).

### 2.5 Evaluation of microvessel count (MVC) and microvessel density (MVD)

All slides were automatically scanned using the virtual slide scanner NanoZoomer (Hamamatsu Photonics) at 40× magnification after data anonymization and setting of image acquisition parameters. The total area of SESCC and adjacent non‐neoplastic esophageal epithelium in one target section was selected, and the total number and area of blood vessels in SESCC and adjacent non‐neoplastic esophageal epithelium, which were stained for both CD31 and α‐smooth muscle actin (α‐SMA), were measured. Further, the microvessel count (MVC) and microvessel density (MVD) were calculated by dividing the total number and area of blood vessels per total area of SESCC or adjacent non‐neoplastic esophageal epithelium, respectively.^31^, ^32^ Representative photomicrographs of double immunohistochemical staining for CD31 and α‐SMA in SESCC are shown in Figure S1A,B.

### 2.6 Oxygen saturation (StO_2_ ) measurement using OXEI

As we previous reported, the mechanism for imaging the oxygen saturation (StO_2_) with OXEI is to detect the difference in absorption coefficient between oxidized and reduced hemoglobin on the mucosal surface using two laser light wavelengths (445 and 473 nm) and process the obtained images and transform it as StO_2_ color maps.^23^ The both images of ordinary white‐light imaging and OXEI imaging with StO_2_ color map can be synchronously captured and filed.

The entire SESCC in pTis‐T1a or the deepest part of SESCC in pT1b, which was captured based on the pathological findings, and the adjacent non‐neoplastic esophageal mucosa were selected with white‐light endoscopic images by two endoscopists. OXEI was synchronized to each target area, and the StO_2_ was quantified using the dedicated software. The StO_2_ difference between SESCC and adjacent non‐neoplastic esophageal mucosa in the same image was calculated as ΔStO_2_. The method used to quantify the StO_2_ and ΔStO_2_ with the OXEI images is shown in Figure S2.

### 2.7 Statistical analysis

Patient and lesion characteristics were summarized using proportion or descriptive statistics such as mean, median, and range. Comparison between groups was assessed by employing Mann–Whitney *U* test. The correlations between the expression of hypoxia markers and MVD were determined using Spearman's rank correlation coefficient. All *p* values were reported as two‐sided, and *p* < 0.05 was considered statistically significant. All statistical analyses were performed with EZR (Saitama Medical Center, Jichi Medical University), a graphical user interface for R 4.1.0 (R Foundation for Statistical Computing). More precisely, EZR is a modified version of R commander (version 2.7‐0) designed to add statistical functions frequently used in biostatistics.^33^

## 3 RESULTS

### 3.1 Clinicopathological characteristics of patients and lesions in pathological studies

Of the 130 lesions from 121 patients, 109 lesions from 102 patients were enrolled and evaluated in this study (Figure S3). The pathological depths of invasion were pTis (36 lesions, 33%), pT1a (42 lesions, 39%), pT1b (31 lesions, 28%). The clinicopathological characteristics of the patients and lesions are presented in Table S2.

### 3.2 The expression of hypoxia markers with immunohistochemical staining by the depth of invasion

**FIGURE 2:**
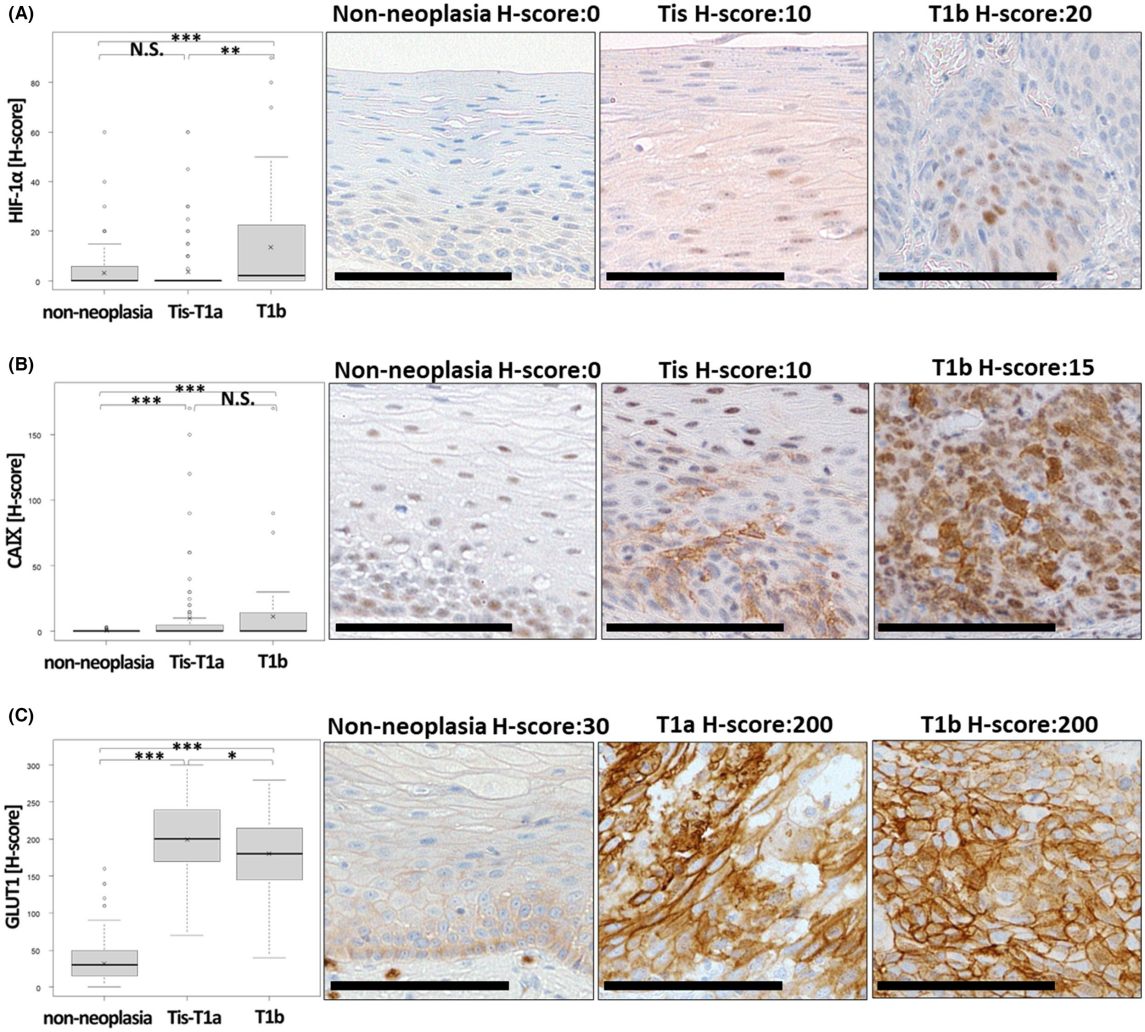
(A, B) Comparison of the expression of hypoxia markers in non‐neoplasia (*n* = 109), pTis‐T1a (*n* = 78), and pT1b (*n* = 31). Additionally, representative photomicrographs of immunohistochemical staining of HIF‐1α (A), CAIX (B), and GLUT1 (C). N.S., not significant, *p < 0.05, **p < 0.01, ***p < 0.001. Scale Bar: 100 μm.

The mean (range) H‐score of HIF‐1α in non‐neoplasia, pTis‐T1a, and pT1b was 4.95 (0–60), 5.51 (0–60), and 16.0 (0–90), respectively. The mean (range) H‐score of CAIX in non‐neoplasia, pTis‐T1a, and pT1b was 0.290 (0–3), 13.0 (0–170), and 15.6 (0–170), respectively. The mean (range) H‐score of GLUT1 in non‐neoplasia, pTis‐T1a, and pT1b was 37.6 (0–160), 199 (70–300), and 177 (40–280), respectively. The expression of HIF‐1α was significantly higher in pT1b than in non‐neoplasia (*p* < 0.001, Figure 2A) and in pT1b than in pTis‐T1a (*p* = 0.00472, Figure 2A). The expression of CAIX and GLUT1 was significantly higher in SESCC than in non‐neoplasia (*p* < 0.001, *p* < 0.001, respectively, Figure 2B,C).

### 3.3 Evaluation of MVC and MVD by the depth of invasion and the relationship between them and the expression of hypoxia markers

**FIGURE 3:**
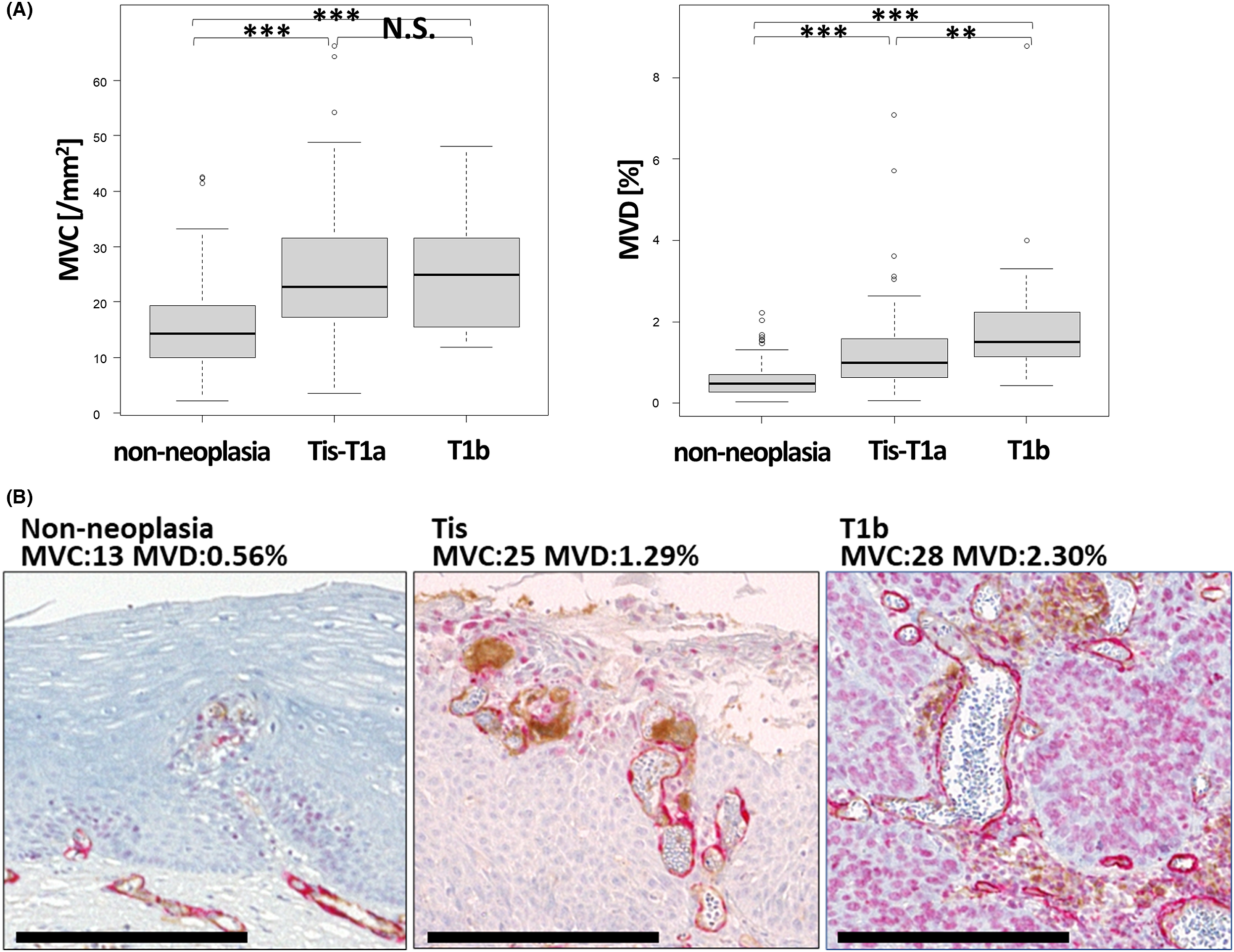
(A) Comparison of MVC and MVD in non‐neoplasia (*n* = 109), pTis‐T1a (*n* = 78), and pT1b (*n* = 31). (B) Representative photomicrographs of MVC and MVD in non‐neoplasia, pTis, and pT1b. N.S., not significant, **p < 0.01, ***p < 0.001. Scale Bar: 500 μm.

**FIGURE 4:**
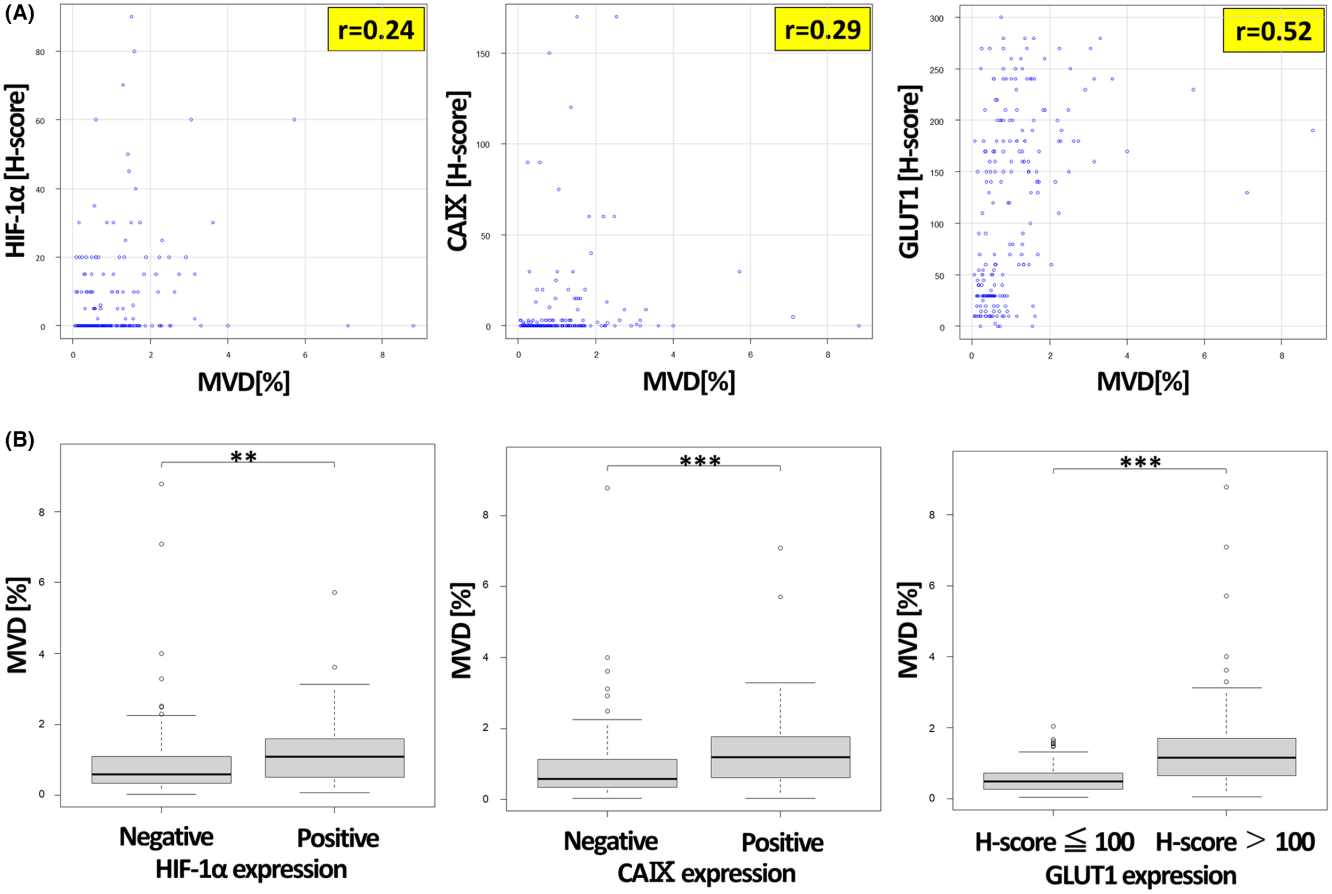
(A) Correlation between MVD and the expression of hypoxia markers in non‐neoplasia and SESCC (*n* = 218). MVD was positively correlated with the expression of HIF‐1α, CAIX, and GLUT1. (B) MVD was significantly higher in the positive expression group of HIF‐1α and CAIX than in the negative expression group, and MVD was significantly increased in the higher expression group of GLUT1 (H‐score > 100) compared with that in the lower expression group of GLUT1 (H‐score ≦ 100). **p < 0.01, ***p < 0.001.

The median (range) MVC in non‐neoplasia, pTis‐T1a, and pT1b was 14.2/mm^2^ (2.14–42.5/mm^2^), 22.7/mm^2^ (3.46–66.3/mm^2^), and 24.8/mm^2^ (11.8–48.1/mm^2^), respectively. MVC was significantly higher in SESCC than in non‐neoplasia (*p* < 0.001, Figure 3A). The median (range) MVD in non‐neoplasia, pTis‐T1a, and pT1b was 0.478% (0.0391–2.24%), 0.991% (0.0639–7.09%), and 1.51% (0.447–8.80%), respectively. MVD was significantly higher in SESCC than in non‐neoplasia (*p* < 0.001, Figure 3A) and in pT1b than in pTis‐T1a (*p* = 0.00479, Figure 3A). Representative photomicrographs of MVC and MVD in non‐neoplastic tissue, pTis, and pT1b are shown in Figure 3B. MVD was positively correlated with the expression of HIF‐1α, CAIX, and GLUT1 (*R* = 0.24, *R* = 0.29, *R* = 0.52, respectively, Figure 4A). Moreover, MVD was significantly increased in the positive expression group of HIF‐1α and CAIX compared with that in the negative expression group (*p* = 0.00120, *p* < 0.001, respectively, Figure 4B) and in the higher expression group of GLUT1 (H‐score > 100) compared with that in the lower expression group (H‐score ≦ 100) (*p* < 0.001, Figure 4B).

### 3.4 Oxygen saturation (StO_2_ ) quantified with OXEI by the depth of invasion

**FIGURE 5:**
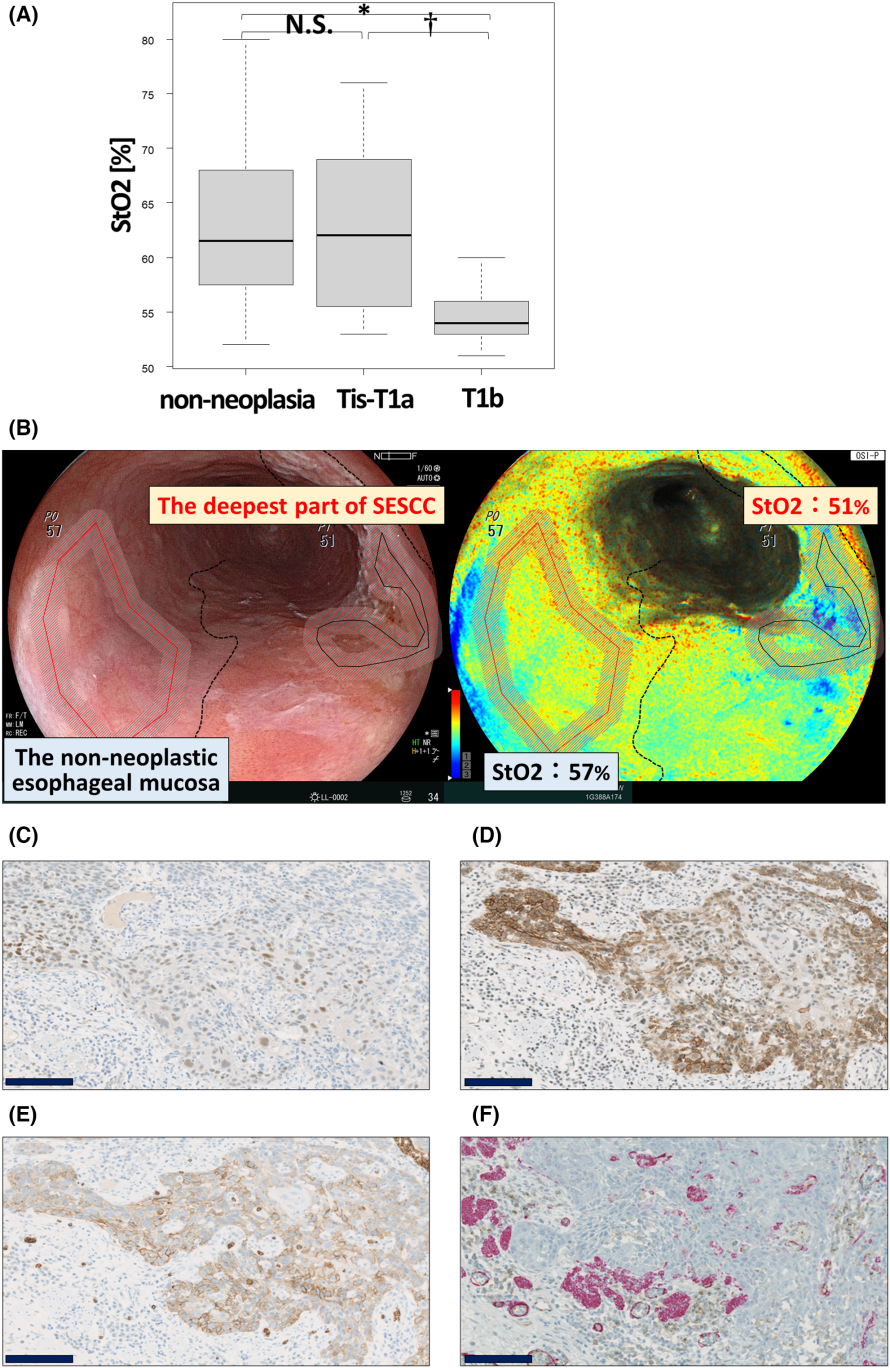
(A) Comparison of StO_2_ in non‐neoplasia (*n* = 16), pTis‐T1a (*n* = 11), and pT1b (*n* = 5). N.S., not significant, †*p* < 0.1, *p < 0.05. (B–E) Representative photomicrographs of StO_2_ quantified with OXEI (B), immunohistochemical staining of HIF‐1α (C), CAIX (D), and GLUT1 (E), and double immunohistochemical staining of CD31 and α‐SMA (F). The StO_2_ in the deepest part of this T1b ESCC was 51% and in this adjacent non‐neoplastic esophageal mucosa was 57%, and ΔStO_2_ was −6%. Scale Bar: 100 μm.

During the study period, we evaluated 16 consecutive lesions from 15 patients both with OXEI before ESD and pathological immunostainings of ESD specimens. The pathological depths of invasion were pTis (5 lesions, 31%), pT1a (6 lesions, 38%), pT1b (5 lesions, 31%). The median (range) StO_2_ in non‐neoplasia, pTis‐T1a, and pT1b was 61.5% (52%–80%), 62% (53%–75%), and 54% (51%–60%), respectively. The StO_2_ was significantly lower in pT1b than in non‐neoplasia (*p* = 0.00131, Figure 5A) and tended to be lower in pT1b than in pTis‐T1a (*p* = 0.0606, Figure 5A). The median (range) ΔStO_2_ in pTis‐T1a and pT1b was −1% (−5% to +3%) and −7% (−13% to −5%), respectively. ΔStO_2_ was significantly lower in pT1b than in pTis‐T1a (*p* = 0.00260, Figure S4). Images of the StO_2_, quantified using OXEI, and photomicrographs of immunohistochemical staining of HIF‐1α, CAIX, and GLUT1 and double immunohistochemical staining of CD31 and α‐SMA in a representative case of pT1b ESCC are shown in Figure 5B–F.

## 4 DISCUSSION

In this study, two important results were obtained. First, the expression of all hypoxia markers and vessel density were significantly different in pT1b compared with those in non‐neoplasia, whereas the expression of HIF‐1α was not significantly different in pTis‐T1a compared with that in non‐neoplasia. Second, the StO_2_ in vivo with OXEI was significantly lower in pT1b than in non‐neoplasia and tended to be lower in pT1b than in pTis‐T1a, whereas it was not significantly different in pTis‐T1a compared with in non‐neoplasia. To the best of our knowledge, this was the first study to compare the expression of hypoxia markers, vessel density, and StO_2_ in non‐neoplasia, pTis‐T1a, and pT1b with ESD specimens. These important results suggest that ESCC becomes markedly hypoxic in especially pT1b.

The strength of this study was the evaluation of the expression of hypoxia markers and vessel density in the largest number of SESCC samples compared with that in previous reports^18^, ^34^ and the inclusion of ESD specimens that were histologically diagnosed as having negative horizontal and vertical margins, excluding surgical specimens, in order to evaluate them under certain conditions. No previous study has examined in detail the oxygen status of SESCC in a large number of cases using only ESD specimens. The expression of hypoxia markers and vessel density were similar as in previous reports, indicating that in ESCC, they become higher as tumor invades deeper.^10^, ^12^, ^13^, ^14^, ^15^, ^16^, ^17^, ^18^, ^35^ When examined in detail, the expression of all hypoxia markers and vessel density were significantly different in pT1b compared with those in non‐neoplasia, whereas the expression of HIF‐1α was not significantly different in pTis‐T1a compared with that in non‐neoplasia, which is a novel finding. In the present study, vessel density was higher in T1b than in pTis‐T1a, suggesting more progressive angiogenesis, similar to previous reports.^18^ It has also been previously reported that the expression of HIF‐1α is higher as angiogenesis progresses.^10^, ^16^ These findings suggest that the expression of all hypoxia markers and vessel density are significantly different in pT1b compared to non‐neoplasia as the oxygen environment becomes hypoxic, caused by the progression of angiogenesis. As a result of less advanced angiogenesis in pTis‐T1a compared to pT1b, the expression of HIF‐1α is almost negligible, suggesting that the oxygen environment in pTis‐T1a may be closer to normoxia than in pT1b.

Further, the strength of this study is that OXEI allowed us to evaluate StO_2_ more safely, in real time, than other methods of evaluating StO_2_. The gold standard method for the quantification of the StO_2_ is the direct measurement of StO_2_ in the tumor using an Eppendorf needle electrode system, but this method is highly invasive because the electrode is directly inserted into the tumor.^36^, ^37^ PET‐based hypoxia imaging with probes such as 18F‐FMISO,^38^ 60 and 64Cu‐ATSM,^39^, ^40^ and 18F‐FAZA^41^ has been reported as a method for non‐invasive estimation of StO_2_, but it has the disadvantage of being affected by drug metabolism and the need to prepare an expensive device. And it must be hard to detect and measure StO_2_ of superficial cancer in digestive tract with PET. OXEI is an image‐enhanced digestive tract endoscopy that can show StO_2_ in vivo, in real time, on digestive tract lesions including SESCC. Similar to ordinary endoscopic observation, OXEI can safely image StO_2_ without puncture and drug administration. In this study, StO_2_ was not significantly different in pTis‐T1a compared to non‐neoplasia, but was significantly lower in pT1b than in non‐neoplasia and tended to be lower in pT1b than in pTis‐T1a, which is a novel finding. The oxygen environment quantified using the Eppendorf needle electrode system and PET has been reported to be hypoxic in some solid tumors compared to non‐neoplasia, and the oxygen environment becomes more hypoxic as they progress.^23^, ^36^, ^37^, ^38^, ^39^, ^40^, ^41^ In a previous study, Kaneko et al^23^ reported that the StO_2_ in vivo with OXEI was significantly lower in esophageal neoplasia than in non‐neoplasia, but it is not known whether StO_2_ was lower in pTis‐T1a or pT1b. Our StO_2_ findings suggest that the oxygen environment is markedly hypoxic in T1b, while it is close to normoxia in pTis‐T1a.

These important results, confirmed by immunohistochemical staining and OXEI, suggest that ESCC becomes markedly hypoxic in pT1b and it may be close to normoxia in Tis‐T1a. This is significant because it clarifies the biological characteristics of the oxygen environment in SESCC. Clinically, pT1b ESCC is markedly more metastatic and has a poorer prognosis than pTis‐T1a.^42^ The present results suggest that factors associated with metastasis may be associated with changes in the oxygen status in SESCC. This may lead to elucidation of the biological mechanisms involved in the malignant transformation associated with submucosal invasion of SESCC. Additionally, OXEI may be useful for the prediction of the depth of invasion between pTis‐T1a and pT1b. Actually, ΔStO_2_ was less than −5 in 1 out of 11 cases with pTis‐T1a and in 5 out of 5 cases for pT1b, and ΔStO_2_ was significantly lower in pT1b than in pTis‐T1a (Figure S4). In SESCC, the prediction of the depth of invasion is important because the treatment strategy differs between pTis‐T1a and pT1b.^43^ One of the imaging methods used to predict the depth of invasion in SESCC is the evaluation of blood vessel morphology by magnifying endoscopy, using narrowband imaging, but its diagnostic capability is not yet sufficient.^43^, ^44^ This result indicates that ΔStO_2_ may be more useful in the prediction of the depth of invasion.

There are some limitations to this study. First, this study was a retrospective study, and we collected as many images as possible, but the number of OXEI cases was small. We are currently collecting the OXEI images of digestive tract lesions including ESCC and hope to investigate larger number of cases in the future. Second, the endoscopically resected specimens included in this study were limited to shallow pT1b ESCC cases that were preoperatively judged to be endoscopically treatable lesions up to the clinical T1b‐SM1. In order to solve this problem, it may be necessary to examine deep pT1b ESCC including the clinical T1b‐SM2/3 with surgery. However, endoscopic and surgical resection may each have different effects on the expression of hypoxia markers and vessel density due to the differences in the procedure of blood flow blockade from major vessels.

In summary, current results suggest that ESCC becomes hypoxic with high vessel density even at an early stage. This is especially prominent in T1b. As the number of cases evaluated with OXEI in this study was small, we would like to increase the number of cases in the future for further studies.

## AUTHOR CONTRIBUTIONS

**Nobuhisa Minakata:** Conceptualization (equal); data curation (lead); formal analysis (equal); funding acquisition (lead); investigation (lead); methodology (equal); project administration (equal); resources (equal); software (equal); visualization (lead); writing – original draft (lead). **Shingo Sakashita:** Conceptualization (lead); methodology (lead); project administration (equal); supervision (equal); writing – original draft (equal); writing – review and editing (equal). **Masashi Wakabayashi:** Formal analysis (lead); methodology (supporting); writing – review and editing (supporting). **Yuka Nakamura:** Resources (lead); software (lead). **Hironori Sunakawa:** Conceptualization (equal); funding acquisition (equal); supervision (equal); writing – original draft (supporting); writing – review and editing (supporting). **Yusuke Yoda:** Conceptualization (equal). **Genichiro Ishii:** Conceptualization (equal); methodology (equal); writing – review and editing (lead). **Tomonori Yano:** Conceptualization (equal); funding acquisition (equal); project administration (lead); supervision (lead); writing – review and editing (equal).

## CONFLICT OF INTEREST STATEMENT

Tomonori Yano received a research grant from Fujifilm Corporation. The Oxygen saturation imaging system is provided by FUJIFILM under research construction. All authors had full access to all the data in the study and are in agreement with the decision to submit this publication; Nobuhisa Minakata, Shingo Sakashita, Masashi Wakabayashi, Yuka Nakamura, Hironori Sunakawa, Yusuke Yoda, and Genichiro Ishii have no conflicts of interest.

## ETHICS STATEMENT

Approval of the research protocol by an Institutional Reviewer Board: This study has been approved by the Institutional Review Board of the National Cancer Center (2020–361).

## Supporting information


Figure S1.

Figure S2.

Figure S3.

Figure S4.

Table S1.
Table S2.

## Data Availability

Not Available.
