# Supplementary figures and images for: Immunohistochemistry and oxygen saturation endoscopic imaging reveal hypoxia in submucosal invasive esophageal squamous cell carcinoma
Source: Cancer Med. 2023 Jun 17;12(15):15809–19. doi: 10.1002/cam4.6217 (PMC10469640; doi:10.1002/cam4.6217)

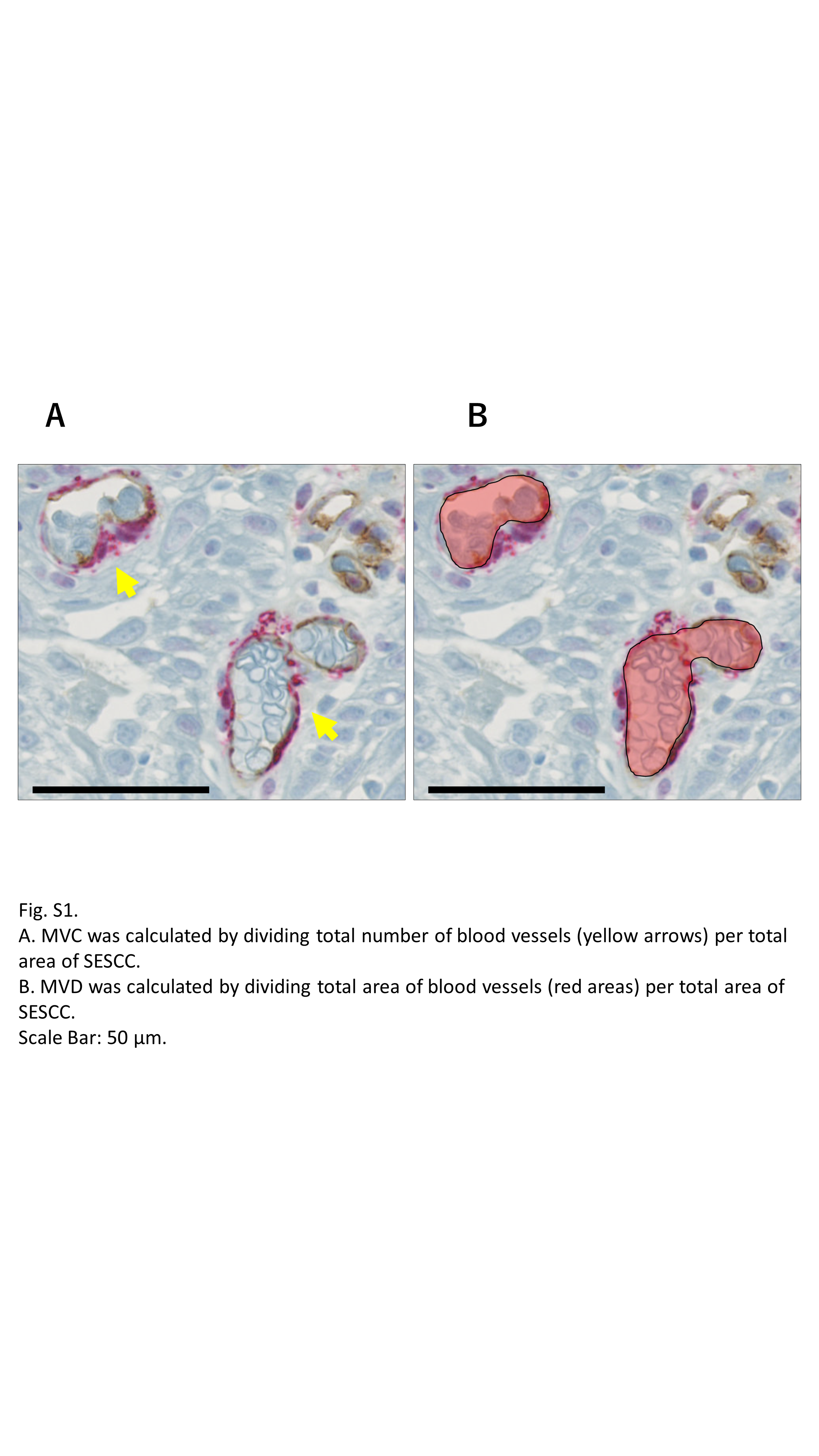

Supplement: Supplementary file 1 — Figure S1. [file CAM4-12-15809-s001.TIF]

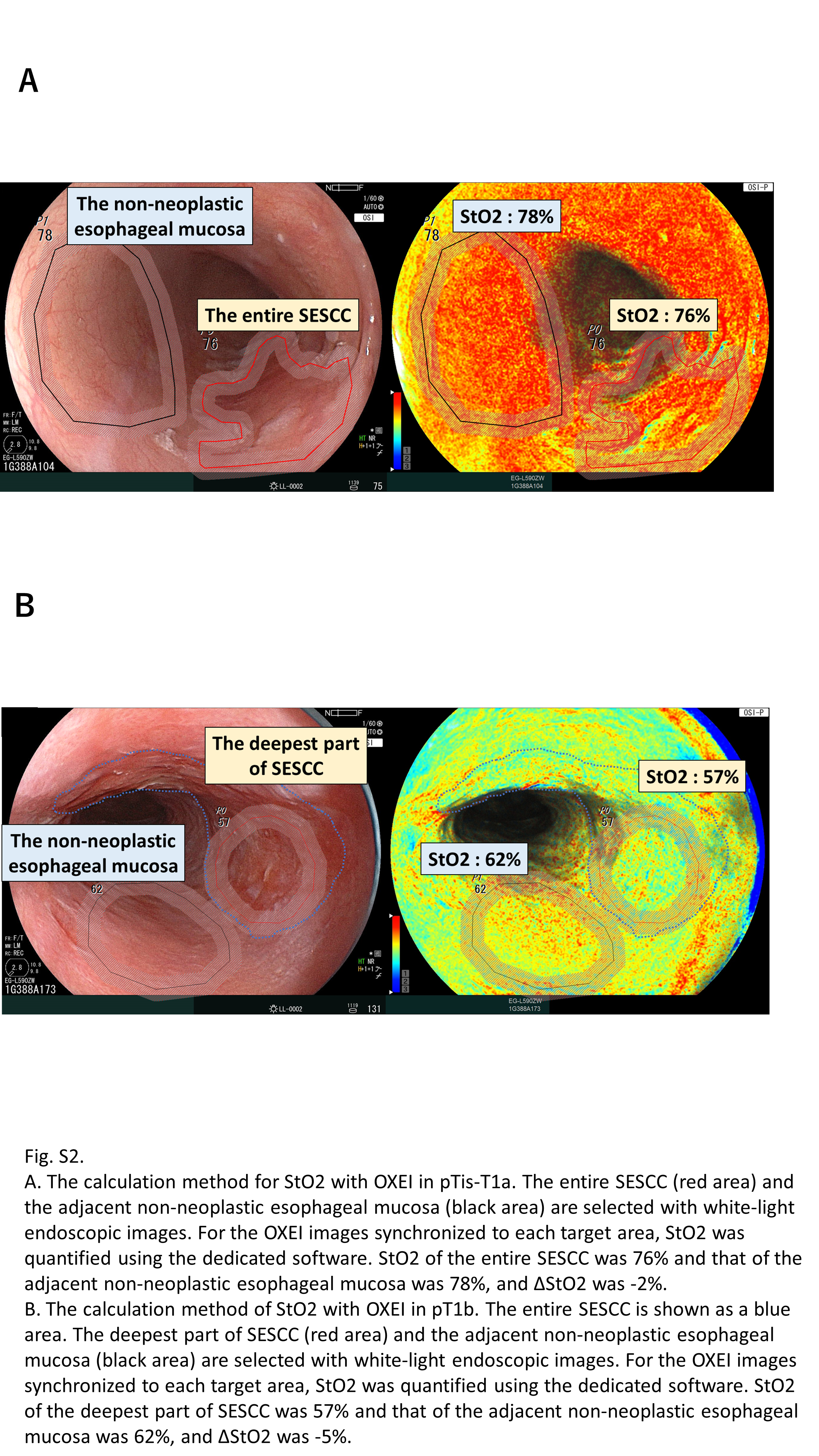

Supplement: Supplementary file 2 — Figure S2. [file CAM4-12-15809-s003.TIF]

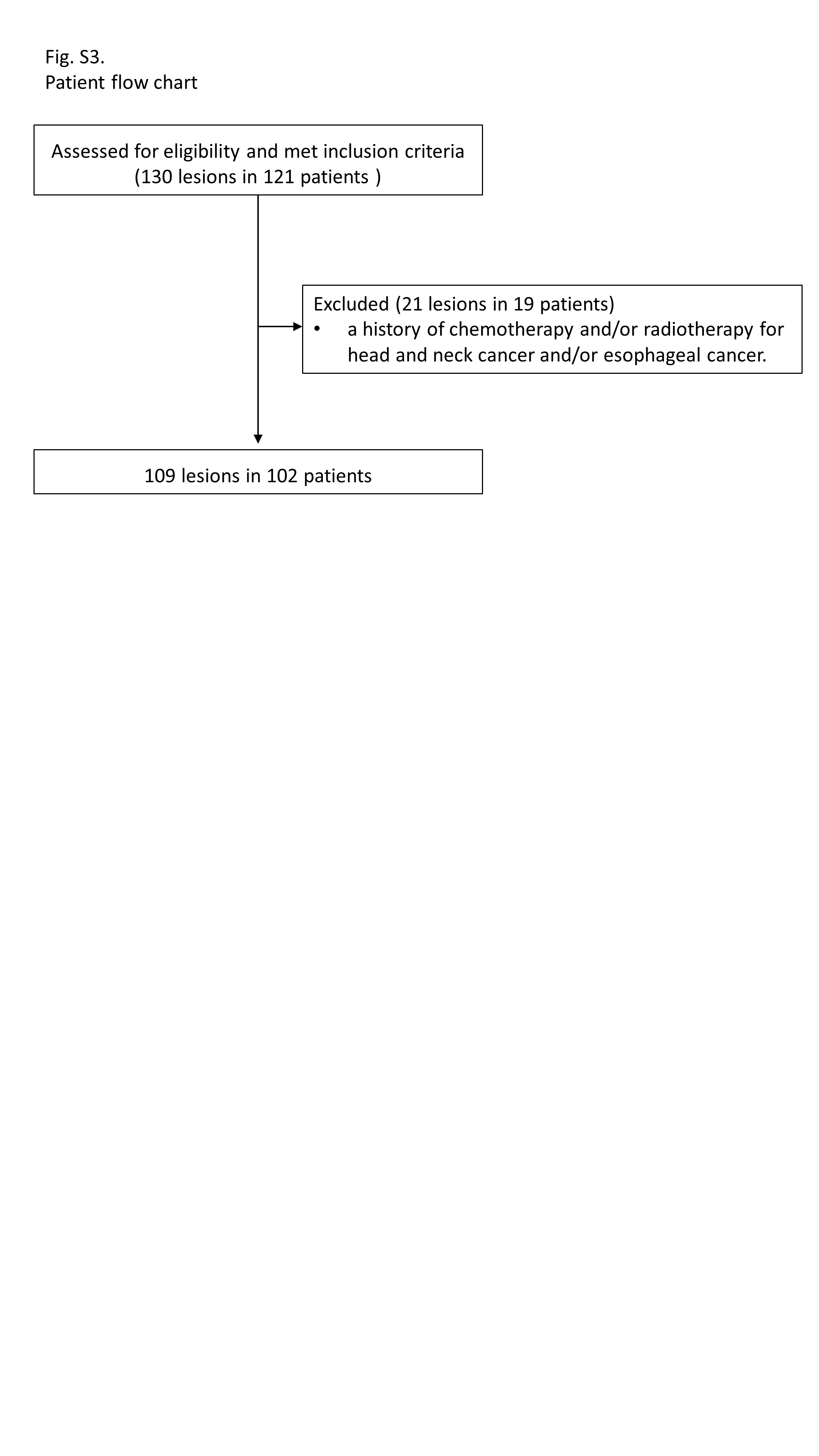

Supplement: Supplementary file 3 — Figure S3. [file CAM4-12-15809-s004.TIF]

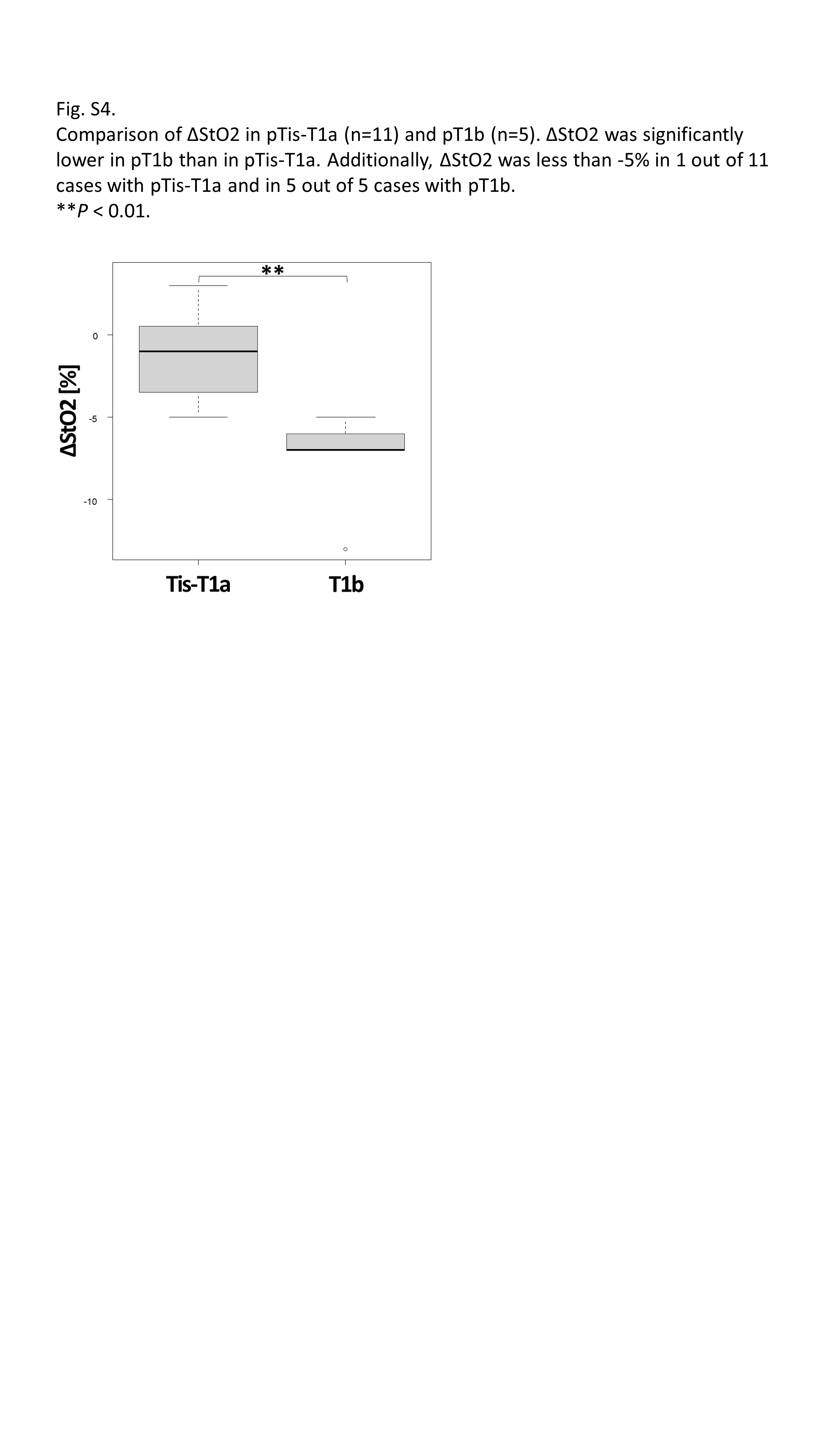

Supplement: Supplementary file 4 — Figure S4. [file CAM4-12-15809-s002.TIF]
