# Supplementary material for: Immunohistochemistry and oxygen saturation endoscopic imaging reveal hypoxia in submucosal invasive esophageal squamous cell carcinoma
Source: Cancer Med. 2023 Jun 17;12(15):15809–19. doi: 10.1002/cam4.6217 (PMC10469640; doi:10.1002/cam4.6217)
Supplement: Supplementary file 5 — Table S1. Table S2. [file CAM4-12-15809-s005.docx]

Table S1.

Primary antibodies used in this study.

| **Primary antibodies** | **Company** | **Species** | **Type** | **Dilution** | **Reference** | **Note** |
| --- | --- | --- | --- | --- | --- | --- |
| **HIF-1α** | Gene Tex | Rabbit | Polyclonal | 1:300 | GTX127309 | at 25℃ incubation for 2 hours |
| **CAIX** | Santa Cruz Biotechnology | Mouse | Monoclonal | 1:300 | sc-365900 | at 25℃ incubation for 1 hour |
| **GLUT1** | Santa Cruz Biotechnology | Mouse | Monoclonal | 1:100 | sc-377228 | at 25℃ incubation for 1 hour |
| **CD31** | DAKO | Mouse | Monoclonal | 1:50 | M0823 | at r25℃ incubation for 36 minutes |
| **αSMA** | DAKO | Mouse | Monoclonal | 1:1000 | M0851 | at 37℃ incubation for 36 minutes |

CAIX, Carbonic anhydrase Ⅸ; GLUT1, Glucose transporter 1; HIF-1α, Hypoxia-inducible transcription factor 1α; αSMA, α-smooth muscle actin

Table S2.

Clinicopathological characteristics of 109 lesions from 102 patients

| Characteristics |  |
| --- | --- |
| Sex, n |  |
| Male | 87 |
| Female | 15 |
| Age, years |  |
| Median | 71 |
| Range | 50-89 |
| Depth of invasion, n |  |
| pTis | 36 |
| pT1a | 42 |
| pT1b | 31 |
| Differentiation, n |  |
| Well | 36 |
| Moderate | 31 |
| Poor, | 11 |
| NA | 31 |
| Lymphatic invasion |  |
| Positive | 15 |
| Negative | 94 |
| Vascular invasion, n |  |
| Positive | 7 |
| Negative | 102 |

pTis, Pathological high-grade dysplasia; pT1a, Pathological intramucosal tumor; pT1b, Pathological submucosal tumor
